# Communicable Disease Surveillance Ethics in the Age of Big Data and New Technology

**DOI:** 10.1007/s41649-019-00087-1

**Published:** 2019-06-10

**Authors:** Gwendolyn L. Gilbert, Chris Degeling, Jane Johnson

**Affiliations:** 1 Marie Bashir Institute for Infectious Diseases and Biosecurity, University of Sydney, Sydney, Australia; 2 Sydney Health Ethics, Sydney School of Public Health, Faculty of Medicine and Health, University of Sydney, Sydney, Australia; 3 Research for Social Change, Faculty of Social Sciences, University of Wollongong, Wollongong, Australia

**Keywords:** Communicable Disease Surveillance, Big Data, Electronic Medical Records, Pathogen Genomics

## Abstract

Surveillance is essential for communicable disease prevention and control. Traditional notification of demographic and clinical information, about individuals with selected (notifiable) infectious diseases, allows appropriate public health action and is protected by public health and privacy legislation, but is slow and insensitive. Big data–based electronic surveillance, by commercial bodies and government agencies (for profit or population control), which draws on a plethora of internet- and mobile device–based sources, has been widely accepted, if not universally welcomed. Similar anonymous digital sources also contain syndromic information, which can be analysed, using customised algorithms, to rapidly predict infectious disease outbreaks, but the data are nonspecific and predictions sometimes misleading. However, public health authorities could use these online sources, in combination with de-identified personal health data, to provide more accurate and earlier warning of infectious disease events—including exotic or emerging infections—even before the cause is confirmed, and allow more timely public health intervention. Achieving optimal benefits would require access to selected data from personal electronic health and laboratory (including pathogen genomic) records and the potential to (confidentially) re-identify individuals found to be involved in outbreaks, to ensure appropriate care and infection control. Despite existing widespread digital surveillance and major potential community benefits of extending its use to communicable disease control, there is considerable public disquiet about allowing public health authorities access to personal health data. Informed public discussion, greater transparency and an ethical framework will be essential to build public trust in the use of new technology for communicable disease control.

## Surveillance and Big Data are Everywhere

Like it or not, we are under constant, state-sanctioned surveillance (Hanley 2017), which is officially “justified” on the grounds of national security, crime prevention, road safety or public service improvement. Unofficially, retailers, goods and service providers and advertisers monitor our preferences, behaviours and habits, for commercial gain—drawing on data provided by us, sometimes voluntarily,^1^ but often unwittingly. Masses of “anonymous” data about population movements, financial transactions and leisure activities are mined, from surveillance cameras, travel cards, smartphones and tablets, wearable devices, internet searches, online orders, credit card use and social media. These data are analysed, compared, integrated and traded without our explicit consent. Surveillance has a long history, but modern technology has revolutionised the accessibility, scope and speed of data collection and analysis.

“Big Data” refers to the rapidly escalating volume, complexity, variety and speed of data acquisition. Big Data analytics is “the process of collecting, organising and analysing large data sets, to discover patterns and generate useful, actionable information” (Garattini et al. 2017). There are risks and benefits associated with Big Data analytics (Davis and Patterson 2012), but little public understanding of what they are, what they depend on and how, if at all, individuals can influence their use. We often assume that data are anonymous, because they do not contain primary identifiers, such as our name or unique (e.g. social security or healthcare) number. Or we are told they have been de-identified, by removal of primary identifiers and other personal data such as address or date of birth. However, experts agree that data de-identification is, at best, provisional because of the plethora of other “anonymous” data and metadata that can be linked, to re-identify individuals (Lubarsky 2017). Even the most sensitive personal data that banks, government agencies, healthcare providers or insurance companies assure us are secure can be accidentally “lost”, deliberately leaked, sold or hacked, with loss of privacy, identity or funds (Tanner 2017).

Big Data is used very effectively in marketing and some types of scientific research, such as meteorology, but relatively little, so far, in healthcare, partly because of privacy concerns and partly because data are often not digital (Bansal et al. 2016). However, health service administrative data, patient medical records and laboratory reports are rapidly being digitised. In the microbiology laboratory, for example, infectious disease research and diagnostics have been transformed by nucleic acid–based pathogen identification and genome sequencing, which are faster, more informative and more amenable to digitisation than traditional culture-based “cottage industry” methods (Gilbert 2002).

In this paper, we discuss the benefits, risks and ethical implications, for individuals and the community, of the intersecting roles of healthcare digitisation, pathogen genomics and Big Data analytics. Less attention has been paid to the application of Big Data to *pathogen* genomics than to its application to *human* genomics but, despite some major differences, there are parallels between them with respect to potential benefits and ethical, legal and social implications (Mattick 2018; Mittelstadt and Floridi 2016; Middleton 2018). We argue that an ethical framework is needed, to guide the use of new technologies in communicable disease surveillance and control.

## Electronic Health Records—Benefits and Risks

Hospitals, general practitioners, diagnostic services, pharmacies, health insurance companies and government healthcare agencies already store personal health data electronically. Most people have several separate records, created by different agencies for different purposes. A universal “cradle-to-the grave” personal electronic health record (EHR), incorporating all personal heath data in a single repository, would have many potential advantages. It would provide immediate access to actionable information in an emergency and could be updated by, and shared among, authorised healthcare providers, as required; it would alert prescribers to drug allergies and interactions, prevent unnecessary investigations and, by the use of personalised decision support systems, provide enhanced diagnostic, therapeutic and prognostic information.

Despite potential benefits, relatively few countries have successfully implemented universal EHRs. Denmark’s system, which is one of the most advanced in the world (Rothstein 2008), has reportedly failed to realise its potential to improve healthcare service delivery, in part because of failure to develop common technical standards for health information exchange (Kierkegaard 2013). Wherever such systems have been proposed, there are unresolved controversies about privacy, effects on doctor/patient relationships and trust, whether individuals can opt out, who controls or can have access to data and when consent is required, how to protect data from unauthorised use or accidental loss (Fairweather and Rogerson 2001), and use of “de-identified” data for ethically approved research. Many of these issues were widely canvassed in recent debates about Australia’s (limited) My Health Record system (Bragge and Bain 2018; Gillespie 2018).

A universal EHR system could have major benefits for healthcare research and delivery. For example, “de-identified” aggregate data can be used to monitor the use, outcomes and quality of health services and inform improvements or generate new knowledge about disease epidemiology, such as spatiotemporal distribution or socioeconomic, environmental or “lifestyle” risk factors. This information would support the development and evaluation of new treatments, preventive strategies or decision support systems and help to address social determinants of disease (Hunter 2018; Marmot 2001). Potential benefits would be attenuated if a substantial proportion of the population were excluded or refused to participate, because of concerns about privacy and data security, or if individual consent were required for access to de-identified data.

Data safety and security cannot be absolutely guaranteed, even with the best technical standards. It is claimed that re-identification is straightforward (Teague et al. 2017) (depending on the de-identification methods used); data can be hacked (BBC World-Asia 2018) and, occasionally, data custodians betray public trust by selling data for financial gain, to pharmaceutical, insurance or software companies (Tanner 2017; Naughton 2017). The risks and potential consequences of misuse are likely to be minimal, if optimal technical standards are applied, but there is often little publicly available information on which to base an informed judgement. Nevertheless, universal EHRs could provide substantial benefits for patient safety and health resource allocation. In the following sections, we argue that linking EHRs to person-specific pathogen genomic data (and/or that of relevant nonhuman animal or environmental pathogens) would enhance the timeliness, precision and effectiveness of public health responses to infectious diseases emergencies.

## Communicable Disease Surveillance and Outbreak Investigations

“Surveillance serves as the eyes of public health” (Fairchild et al. 2007) or “the finger on the pulse of the health of a community” (Lee et al. 2012). The WHO defines surveillance as the “…systematic ongoing collection, collation and analysis of data for public health purposes and the timely dissemination of public health information for assessment and public health response as necessary”(WHO, n.d). Communicable disease surveillance dates back to, at least, the nineteenth century. Its purpose is to identify and provide appropriate care of people affected by diseases of public health importance and their immediate contacts; prevent the spread of disease; and detect, investigate and control outbreaks. Recent infectious disease outbreaks and pandemics have demonstrated its continuing importance (Box 1).

Box 1 Detecting exotic or emerging infections of high consequence

**Table:** 

In late February 2003, a man, who had recently travelled to China, presented to a hospital in Hanoi with what an astute clinician recognised as an unusual, severe acute respiratory syndrome (which would come to be known as SARS). At about the same time, the Chinese Ministry of Health announced, belatedly, that an outbreak of severe atypical pneumonia in Guangdong province had already claimed at least 300 lives, since November 2002. Scientists and epidemiologists, from WHO’s Global Influenza Surveillance (GISN) and Global Outbreak and Response (GOARN) networks, immediately began collecting and analysing microbiological, clinical and epidemiological data. By mid-March, another 150 suspected cases of SARS had been identified in Hong Kong, Singapore, Vietnam and Canada (Heymann and Rodier 2004). Despite China’s delayed outbreak report, a massive global effort, led by WHO and GOARN, rapidly identified a novel coronavirus (SARS CoV) as the cause. They documented modes of transmission, nosocomial infections, risk factors and a high mortality, which enabled WHO to develop evidence-based guidance for diagnosis, management, hospital infection control, quarantine and travel. Within 6 months, the global spread of SARS had ceased, albeit only after it had spread to 29 countries on six continents, caused 8437 cases (of which 92% were in China) and 813 deaths, and cost the global economy an estimated US$54 billion (Knobler et al. 2004). Similar delays in recognition and reporting of the 2013–2014 Ebola virus disease outbreak in West Africa led to unprecedented cross-border transmission and, ultimately, > 28,000 cases and 11,000 deaths—mostly in the three affected countries—before it was eventually brought under control by a massive, coordinated international effort (Koch 2016a).	

These outbreaks illustrate not only the serious risks for the source countries, of inadequate surveillance and delayed outbreak detection, but also the benefits of prompt public health intervention once outbreaks have been recognised. The SARS (2003) and H1N1 influenza (2009) pandemics highlighted the importance of effective communicable disease surveillance, for national and international health security. The WHO International Health Regulations (IHR) (WHO 2008) provide a legal framework for disease detection and response. Many countries still do not comply with the IHR, but WHO has increased their efforts to encourage and support implementation, especially in low- and middle-income countries. In most high-income countries, communicable disease surveillance has been in operation for decades and credited with rapid detection of novel disease incursions, such as Hendra virus in Brisbane, 1994 (Selvey et al. 1995), and West Nile virus in New York, 1999 (Sejvar 2003), and national monitoring of pandemic influenza 2009 (NSW Public Health Network 2009) as well as outbreak control. Methods used range from reports of unusual or suspicious index cases by astute (medical or veterinary) clinicians to mandatory notification of diseases of public health importance, syndromic surveillance and digital epidemiology.

## Notification of Communicable Diseases of Public Health Importance

Notification of communicable diseases, to central public health authorities, allows coordinated public health action. Detection and investigation of outbreaks require prompt, accurate laboratory diagnosis and follow-up of affected individuals. This means that personal information—names, ages, addresses and relevant medical data—is reported to public health authorities, often without the person’s knowledge or consent. Patients and their contacts are questioned to determine the likely sources and the extent of the outbreak. Patients are treated, if necessary, and may be isolated, while they remain infectious, to prevent further spread; contacts, or occasionally whole communities, may be quarantined (Koch 2016b), especially if preventive measures, such as vaccination or antibiotic prophylaxis, are unavailable.

These interventions are intrusive and have been a source of controversy, since the late nineteenth century, when physicians objected to tuberculosis surveillance, which they claimed would encroach on the sanctity of the patient-doctor relationship (Shrady 1897). However, throughout most of last century, there has been widespread public acceptance of name-based communicable disease surveillance of selected (prevalent, serious and/or preventable) infectious diseases, based on a generally well-founded assumption of privacy—i.e. that information will be conveyed only to those who need to know. The benefits include development of evidence-based infection prevention, disease control and health service planning strategies and the ability to monitor disease epidemiology (Fairchild et al. 2017) and inform development of vaccines and antimicrobials.

Collection and storage of personal data for communicable disease control are usually protected by public health and privacy legislation. Until recently, it has been relatively inefficient. Conventional paper-based disease notification, by mail or fax, and “shoe-leather” outbreak investigations are slow. Hard copy records are “protected by chaos” (Rothstein 2008) and difficult to access for unauthorised, inappropriate or even legitimate use, such as approved research. Culture-based laboratory diagnosis and referral of isolates to a reference laboratory for strain typing takes days or weeks. By then, important epidemiological information (such as food history) is often lost or forgotten and the outbreak is likely to have spread. Recently, faster, more accurate pathogen identification and strain typing methods, including whole-genome sequencing (WGS) (Köser et al. 2012a), and automated laboratory reporting have improved the timeliness of outbreak detection and provided more accurate microbiological information for public health action.

## Outbreak Management Using Pathogen Whole-Genome Sequencing

Over the past 20 years, increasingly sophisticated genotypic methods have been developed for pathogen strain typing but, until recently, most have been expensive, time-consuming and/or not particularly discriminatory and mainly used for retrospective outbreak investigation. However, newer strain typing methods have been used routinely, for several years, to improve detection of outbreaks due to, for example, *Mycobacterium tuberculosis* (Merker et al. 2017)*,* foodborne pathogens, such as *Salmonella* Enteritidis and its numerous serovars (Campioni et al. 2015), and nosocomial pathogens such as methicillin-resistant *Staphylococcus aureus* (O’Sullivan 2006). Pathogen whole-genome sequencing (WGS) is the ultimate strain typing method. Recently, it has and is continuing to become faster, less expensive and more informative than other genotypic methods and is being introduced into routine use in public health laboratories (Ashton et al. 2016; Satta et al. 2017; Inns et al. 2017; Gurjav et al. 2016). It promises to dramatically improve the accuracy and speed of pathogen identification, antimicrobial resistance (AMR) profiling, biological risk prediction, outbreak identification and pathogen tracking (ECDC 2016; Quainoo et al. 2017), with definitive discriminatory power.

The value of WGS lies in the fact that microbial genomes change over time due to point mutations of nucleotides—i.e. single nucleotide polymorphisms (SNPs)—which occur at different rates between species and between different regions of the genome.^2^ Epidemiologically related isolates (i.e. from the same outbreak) are identical or different from each other by small numbers of SNPs, whereas differences between unrelated isolates are much larger. As the pathogen spreads from person to person, differences between the outbreak isolates’ genomes increase, as SNPs accumulate, but remain small, compared with those between unrelated genomes. By comparing genomes of outbreak isolates with each other and with a reference strain of the same pathogen, one can infer the approximate date of onset and sequence of transmission events (Dudas et al. 2017), from the number and positions of SNPs. WGS of related isolates can confirm (or exclude) individual transmission events (Arnold et al. 2016), reveal who infected whom, whether there are gaps in the order of transmission (Köser et al. 2012b), which may indicate unrecognised cases or asymptomatic carriers. If relevant isolates are available, it can identify an index case or common source (e.g. food) of widely dispersed cases (Inns et al. 2017), and it can identify “superspreaders” (Stein 2011)—individuals who infect a disproportionately large number of other people.

Some of this information can be discovered by traditional epidemiological investigation, but generally, only much more slowly and with greater difficulty. People may not know, may forget or not wish to reveal what, where or with whom they ate or were in contact, days or weeks before. Implicating a place (restaurant, food processing plant, farm), person or animal as the source of an outbreak has potentially serious medico-legal, commercial or international trade implications (Stasiewicz et al. 2015; Lüth et al. 2018) and genomic data are more objective and, hence, more convincing than epidemiological data. Nevertheless, these different types of information are complementary and both are needed to validate results.

Additional technical developments are needed before the benefits of WGS can be fully realised, but they are feasible or already in progress. They include greater automation and standardisation of quality control methods, bioinformatics tools and algorithms, for analysis and interpretation, and networking of laboratory databases to allow real-time monitoring for outbreak detection (Kwong et al. 2015).

### Implications and Risks of WGS for Surveillance and Outbreak Investigations

Surveillance and outbreak investigation has always required the use of personal data. The much greater precision of WGS raises new questions of consent and unanticipated harm, but despite its increasing use in public health and hospital laboratories (Quainoo et al. 2017; Azarian et al. 2015), there has been little discussion of these issues. Unlike public health surveillance, nosocomial outbreak investigations are not protected by legislation and there is no clarity about privacy protection or the need for informed consent when the use of WGS is extended into new domains. In the following case study (Box 2), we outline some ethical issues raised by WGS of stored isolates for retrospective investigation of a hospital outbreak and suggest that, while the benefits would have been greater if WGS had been available at the time, the ethical dilemmas would have been even more challenging.

Box 2 A high stakes neonatal intensive care unit outbreak investigation^3^ (Pinto et al. 2013)

**Table:** 

Two very premature infants aged 9 and 11 days, respectively, who were nursed in the same room of a neonatal intensive care unit (NICU), developed fulminating methicillin-resistant *Staphylococcus aureus* (MRSA) sepsis and died within 2 days of each other, despite appropriate treatment. Routine genotyping showed they were infected with the same rare MRSA strain. Possible sources were vaginal colonisation of one mother, transmitted to her infant at birth and then to the other infant; or unrecognised colonisation of a patient, staff member or visitor transmitted, by direct or indirect contact, to both infants. NICU staff and patients were screened for MRSA colonisation. Although screening of staff is controversial and rarely indicated, they readily agreed, after being assured of confidentiality. Routine admission and weekly follow-up screening of new inpatients was implemented. No patient or staff member was identified as carrying the MRSA outbreak strain by initial screening, but over the next 7 months, 13 additional infants became colonised, indicating that nosocomial transmission was continuing, despite enhanced infection control measures. Several of these infants were already colonised with the MRSA outbreak strain within hours of delivery, by caesarean section. Therefore, operating suite and NICU staff (again) were screened. One colonised NICU staff member was assumed to have acquired it from a colonised infant, whom she was nursing and no one was identified as a likely source. However, soon after this, another NICU staff member attended the hospital emergency department, with an infected leg abrasion, from which the MRSA outbreak strain was isolated. She was treated and returned to work. Subsequently, for several months, no newly MRSA-colonised infants were identified; the outbreak was apparently over. This raised the possibility that this latter NICU staff member had been the an unwitting source or vector of ongoing transmission. Her screening swabs had been negative, but sites of MRSA colonisation other than the nasal mucosa (the only site swabbed) are not uncommon. The outbreak strain reappeared in the NICU 7 months later and, subsequently, was isolated from patients in the emergency department and other hospital wards, most of whom had some contact with the NICU. WGS was not available at the time of the outbreak, but it was performed, 5 years later, on stored MRSA outbreak isolates to determine, if possible how this unusual, highly virulent MRSA strain was introduced. What/who were the source and/or vector(s) of continued transmission? Was the reappearance of the outbreak strain, after seven months, due to ongoing transmission or a new introduction?	

WGS of stored outbreak isolates, for retrospective outbreak investigation, is likely to provide some answers to epidemiological questions, but before it is done, several important ethical questions should be considered:

- Should informed consent for WGS be obtained from individuals from whom isolates were obtained (or their carers), despite logistical difficulties and emotional risks? Individual consent was given, at least implicitly, for collection of specimens for diagnosis or screening and whatever laboratory procedures were in routine use at the time, but WGS is a new procedure, and its results will have potentially significant implications, for affected individuals.
- If WGS identifies an individual as a likely source or vector of nosocomial transmission, how would this reflect on her infection control practices or affect her future employment? What would be the psychological effects of discovering that she had transmitted a pathogen to vulnerable patients in her care (even if no blame were attributed to her by others)? Should she be told?
- What are the potential medico-legal implications for the hospital, if a staff member were identified as a vector of pathogen transmission? Transient contamination of other staff members’ hands was likely but unverifiable.
- Who owns microbial isolates and the information they contain—the people from whose clinical samples they were isolated or the laboratory, which isolated and characterised them?

Questions like these are even more relevant now that the use of WGS is increasing, not only for research or retrospective outbreak investigation but, potentially, for routine pathogen identification, AMR testing and prospective hospital infection control (O'Sullivan et al. 2012).

### Use of Pathogen WGS and Metagenomics for Routine Diagnosis and Strain Typing

It is predicted that WGS will replace conventional culture-based pathogen identification and AMR testing, in public hospital and private diagnostic laboratories, within a few years (Kwong et al. 2015). Pathogen identification and sequencing, directly from clinical specimens, without the need for culture or prior knowledge of the target pathogen (clinical metagenomics^4^), is likely to be possible in the future. When it is, sequence-based pathogen identification and AMR testing results will be available within hours. This will allow appropriate antimicrobial therapy for a bacterial or fungal infection, to be started much sooner than is currently possible or avoided altogether, if a viral infection is identified. Either way, outcomes will be better, with fewer drug side effects, improved antimicrobial stewardship, less AMR and less frequent pathogen transmission. There are still major barriers to routine culture-based WGS or culture-independent metagenomics, but decreasing costs, faster sequencing, the potential to identify virulence or AMR markers and polymicrobial infections and/or to predict outbreaks with a single assay, make them attractive options for clinical microbiology (Forbes et al. 2017; Gardy and Loman 2018).

### Integrating Genomic, Clinical and Epidemiological Data

Human genomic data and Big Data analytics are already being combined for personalised diagnosis and treatment (“precision medicine”) of some diseases (Brittain et al. 2017). The use of pathogen WGS or metagenomic data will expedite the introduction of precision medicine in infectious diseases. Application of Big Data analytics to (i) demographic, clinical and lifestyle risk factors (from EHR); (ii) genetic and immunological predictors of risk and response to therapy (from personal genomic data); and (iii) pathogen virulence and AMR profiles (from WGS data) would allow individually tailored antimicrobial therapy (drug, dose, route of administration, duration) and improved outcomes.

Infectious diseases differ from most others in that the speed of diagnosis and time to initiation of appropriate therapy (within hours, for life-threatening sepsis) is the major determinant of the outcome, not only for the affected individual but also for her immediate contacts and the wider community. Rapid identification is obviously essential to ensure prompt, appropriate care and isolation of a patient with a potentially fatal meningococcal or an imported pan-resistant Gram-negative bacterial infection; extremely drug-resistant tuberculosis; or an exotic, high impact viral infection, e.g. Ebola or pandemic influenza. It is also important to allow timely application of appropriate hospital infection control and public health interventions, such as vaccination, antimicrobial prophylaxis and/or quarantine of contacts, to prevent or limit a dangerous outbreak.

Many benefits for individual patients and their (known) contacts could be achieved by the use of individual EHRs, alone, but personalised treatment regimens and outcome monitoring also depend on the use of algorithms derived from analysis of aggregate data from large numbers of EHRs. Big Data analytics can not only develop but also validate and improve diagnostic, treatment and decision support algorithms and, potentially, identify previously unrecognised prognostic information such as clinical or genetic markers of susceptibility to infection, an excessive immunological response or of being a “superspreader”. Thus, the community benefits of an EHR system, for disease prevention and research, would depend on widespread—ideally universal—community participation (not unlike vaccination).

Moreover, to realise the additional benefits of using pathogen WGS data for communicable disease surveillance will require access to and analysis of aggregate WGS data in national or international laboratory networks, linked to clinical and epidemiological databases. EHRs of individuals, whose isolates are identified as part of an outbreak, would be scanned for common risk factors, contacts or exposure to environmental sources, of which the individuals, themselves, may be unaware, to expedite outbreak investigation. Automatic access to, at least, selected personal EHR data, without the need for individual consent, will be essential, since the effectiveness of public health interventions depends on speed. However, data linkage could disclose information that individuals may want to remain private. The trade-off has implications for the effectiveness of surveillance systems because, as “utility increases privacy decreases” (Lubarsky 2017). The challenge is to ensure an appropriate balance between individual risk and community benefit.

## Syndromic Surveillance, Digital Epidemiology and Big Data

The use of pathogen WGS or metagenomics for surveillance, as outlined above, is highly specific, but also selective and insensitive. Infected (and infectious) people, who are asymptomatic or have mild symptoms, who do not seek or have access to medical care or for whom laboratory tests are not ordered, may unwittingly spread infection to others. Syndromic surveillance can “capture” undiagnosed infections and is commonly used to complement other methods. One important source of syndromic data is medical encounters such as emergency department or office visits (Henning 2004; Muscatello et al. 2005). Automated monitoring of coded, de-identified data, analysed in almost real-time, allows health authorities to rapidly identify the onset, trace the spread and identify trends, over time, of outbreaks, e.g. of diarrhoeal or respiratory diseases; it can provide early warning of seasonal, or even pandemic, influenza, or track adverse reactions to antimicrobial drugs or vaccines (Salathé 2016). Although syndromic surveillance contains high levels of background “noise”, its strengths are timeliness and pattern recognition.

Digital epidemiology refers to the study of disease patterns using digital data (Gardy and Loman 2018; Salathé et al. 2012). For communicable disease epidemiology, data may include conventional syndromic surveillance, as outlined above, as well as, for example, participatory surveillance systems, to which volunteers report symptoms online (Guerrisi et al. 2016); calls to hospital or nurse help lines; ambulance dispatch requests; health insurance claims; and laboratory, pharmacy or EHR records. They can also include any of the myriad data collected for unrelated purposes that may (or may not) reflect disease activity, such as school and work absenteeism rates, over-the-counter drug sales, social media posts (Salathé et al. 2012; Charles-Smith et al. 2015; Tang et al. 2018) and internet searches (Ginsberg et al. 2009). Data from animal and environmental health sources can help to identify emerging infectious disease risks.

Applying Big Data analytics to diverse and underutilised data, often from otherwise hidden populations, could enhance outbreak detection and our understanding of infectious disease epidemiology on a global scale, rather than the local or national focus of conventional surveillance. The greater scope, diversity and geographic range of data sources would increase the potential for acquisition of new knowledge, modelling of disease outbreaks, trends and related human behaviour and, ultimately, for improved global control, reduced morbidity and mortality from communicable diseases and greater health security. But there are also potential, largely unknown and poorly defined, risks.

One risk is the use of flawed methods for disease prediction or modelling. The accuracy and reliability of different types of data, and the methods used to analyse them, are highly variable, e.g. references to “flu”, on Facebook, “flu-like illness” in an experienced GP’s case notes or “influenza A virus identified” in a laboratory report could apply to the same, or to very different, conditions. The limitations of novel surveillance systems were illustrated by the demise of Google Flu Trends, which was based on Google searches related to “flu” or symptoms or behaviours interpreted as likely to be due to flu. It was predicted, but ultimately failed, to identify the onset of seasonal influenza 2 weeks in advance of conventional surveillance (Ginsberg et al. 2009; Lazer et al. 2014). Its failure was attributed to the lack of transparency of methods, over-fitting of data and failure to account for changing search behaviours (Lazer and Kennedy 2015).

Another risk is that many data sources do not capture basic demographics (age, sex, ethnicity) and infants, the elderly and economically disadvantaged groups—who are most at risk from communicable diseases—are likely to be under-represented (Bansal et al. 2016). Methods developed to minimise bias and validate results in conventional epidemiological research, such as sampling protocols and case definitions, cannot easily be applied (Lee et al. 2016).

The harmful effects, of inaccurate or exaggerated outbreak predictions or modelling, include economic impacts on trade, tourism and health services, social consequences of unnecessary public fear and loss of trust in public health authorities. Appropriately skilled, multidisciplinary development teams could anticipate and mitigate these risks. Peer review and ethical oversight of methods and validation of results by comparison with conventional data would help to prevent them—albeit at increased cost of program development and maintenance.

Perhaps, the greatest risk, however, is public fear, ignorance and mistrust. Ill-informed media scrutiny and political risk aversion could prevent or delay the incorporation of de-identified personal health data into Big Data–based public health surveillance, despite the benefits. Including them would complement and help to validate less-specific, less-reliable data and mitigate the risks. Even the best available de-identification methods, and optimal levels of data security around legitimate use of identified personal data for outbreak investigations, may not placate these fears. However, well-designed public education and consultation initiatives, supported by suitable privacy regulation and regulation of standards, would improve trust. On the other hand, if important data were, theoretically, available but omitted, public health authorities would attract public, media and political censure if they failed to prevent or limit an infectious disease emergency because they failed to utilise or respond to intelligence, whatever the source, which could have predicted it.

There is an urgent need for open and informed discussion about the ethical implications, quality and safety of Big Data–based use of varied types of data, including personal and pathogen genomic data, in communicable disease control. Questions for discussion—assuming the most reliable methods of data capture and analysis are in use—might include, but not be limited to:

- What is the personal significance of a pathogen isolated from an individual, considering the highly specific and sensitive personal information that its WGS can reveal? Should it be subject to a similar degree of privacy protection as that of her personal (human) genome?
- Given the public benefits (accuracy, reliability, sensitivity, specificity, timeliness) of using as many and varied types of data as possible, in communicable disease surveillance, outbreak investigation and research, should the use of de-identified personal data from EHRs (which may include personal and/or pathogen genomic data) be permitted, and in what circumstances? Do individuals have a moral obligation to participate? Should individual consent be required and if so in what circumstances?
- What level of probability and reliability, based on Big Data analytics, should be required before issuing a public alert about an impending infectious disease emergency—e.g. due to a previously unknown emerging or highly virulent, transmissible or drug-resistant pathogen—considering the need to balance the risks of delay against those of a false alarm? (Would a 30% risk of a dangerous infectious disease emergency be *morally* equivalent to a 30% risk of a category 4 tropical cyclone, for example?)
- What would be an ethical response to information, based on reliable Big Data analytics that some populations are at increased risk of infection and/or of becoming a risk to others? Considering the risk of stigmatisation, discrimination, loss of autonomy (e.g. mandatory testing) and/or restriction of liberty (e.g. quarantine), who should be told (the affected group; health professionals; the general public)?

Answers to these and other questions would inform development of an ethical framework, for future communicable disease control, which, we argue, is needed because recent technological innovations have raised new ethical issues. A framework would guide development of policies to optimise benefits and minimise risks, protect vulnerable populations and build public trust in and support for public health action in an infectious disease emergency.

## Footnotes

1 Users of online applications consent to the collection and use of their data by accepting “Terms and Conditions” as a condition of use, but the small print is so long and detailed that most of us do not read it or understand the implications.

2 Bacterial genomes also change by addition or deletion of blocks of DNA. This complicates but does not invalidate interpretation of WGS. Most of what follows refers to bacterial genomes to which WGS has been most recently applied, but the principles apply to all microbial pathogen.

3 This case study is modified from an actual outbreak report (Pinto et al. 2013)

4 Culture-independent pathogen identification, using nucleic acid detection such as polymerase chain reaction (PCR) has been used for years, but has not replaced culture, when the likely pathogen(s) is/are uncertain or AMR testing and/or strain typing are required. Metagenomics allows unbiased, detection and sequencing of all micro-organisms in a sample, including, potentially, unexpected, rare, novel, unculturable and/or multiple pathogens.
